# Tumor-to-tumor metastases: papillary thyroid carcinoma into a clear cell renal cell carcinoma

**DOI:** 10.1186/s40463-017-0193-3

**Published:** 2017-03-01

**Authors:** Jin Soo Andy Song, S. Mark Taylor, Jonathan Trites, Matthew H. Rigby, Martin Joseph Bullock, Jennifer Merrimen, Ricardo Rendon, Robert D. Hart

**Affiliations:** 1 Division of Otolaryngology–Head & Neck Surgery, Dalhousie University, Halifax, Nova Scotia Canada; 2 Department of Pathology, Dalhousie University, Halifax, Nova Scotia Canada; 3 Department of Urology, Dalhousie University, Halifax, Nova Scotia Canada

**Keywords:** Tumor-to-tumor, Papillary thyroid carcinoma, Clear cell renal cell carcinoma

## Abstract

**Background:**

Thyroid metastases to distant sites are uncommon incidents, most often metastasizing to the lungs and bones. Rates of metastasis to the kidney are particularly low, ranging from 2.8–3.8% for papillary and 6–20% for follicular variants of well-differentiated thyroid cancers (WDTCs). In rare instances, tumor-to-tumor metastasis between two true primary neoplasms can occurs. This medical phenomenon has previously occurred as a clear cell renal cell carcinoma (CCRCC) spreading to a WDTC. To our knowledge, this is the first report of a tumor-to-tumor metastasis of a thyroid cancer metastasizing to a primary renal neoplasm.

**Case presentation:**

A 72 year old male presented to the urology clinic with complaints of flank pain. Computed tomography (CT) imaging of the abdomen and pelvis revealed a 5.7 cm solid enhancing mass from the lateral aspect of the right kidney, suspicious for renal cell carcinoma (RCC). The patient subsequently underwent a right laparoscopic radical nephrectomy, and immunohistochemical staining of the 5.5 cm lesion revealed a positive RCC marker to establish a diagnosis of a pT1b ISUP Grade 2 CCRCC. The tumor contained a 3 mm focus of a lesion staining positive for TTF1 and Thyroglobulin, and negative for RCC marker. This finding established a diagnosis of a tumor-to-tumor metastasis of PTC to CCRCC. Subsequent ultrasound and CT of the head and neck revealed a heterogeneously hypodense 3.3 cm mass in the right thyroid lobe, prompting a total thyroidectomy and level VI neck dissection. Pathology revealed a classic variant multifocal PTC and two ipsilateral lymph nodes positive for metastatic PTC. Ultimately, the thyroid specimen was positive for lymphatic vascular invasion, extrathyroidal extension with invasion of the tracheal cartilage, staging as T4aN1aM1. On follow up examination the patient was recovering well, without signs of dysphagia or dysphonia, and showed bilateral mobile vocal cords on laryngoscope examination.

**Conclusions:**

Tumor-to-tumor metastasis between the thyroid and kidney is an extremely rare occurrence, reports of RCC metastases from a WDTC has not yet been reported in the literature. Corroboration of diagnostic imaging findings with immunohistochemistry staining can consolidate a diagnosis of thyroid neoplasm tumor-to-tumor metastasis to a RCC, thereby prompting surgical excision.

## Background

Thyroid metastases to distant sites are relatively uncommon, most often metastasizing to the lungs and bones [1]. Rates of metastasis to the kidney are particularly low, ranging from 2.8–3.8% for papillary and 6–20% for follicular variants of well-differentiated thyroid carcinoma (WDTCs) [2]. Papillary thyroid carcinomas (PTC) are the most common presentation of thyroid malignancy at 80%, with classic-PTC being the most common subtype [1]. In extremely rare instances, tumor-to-tumor metastasis occurs. The criteria for tumor-to-tumor was first defined by Campbell et al. in 1968 as two true primary neoplasms, with the donor tumor metastasizing into the substance of the host tumor [3]. This phenomenon has been shown to occur between a WDTC and a clear cell renal cell carcinoma (CCRCC), in which the CCRCC spreads to the WDTC. To our knowledge, this is the first report of a tumor-to-tumor metastasis of a thyroid cancer metastasizing to a primary renal neoplasm.

## Case presentation

**Fig. 1:**
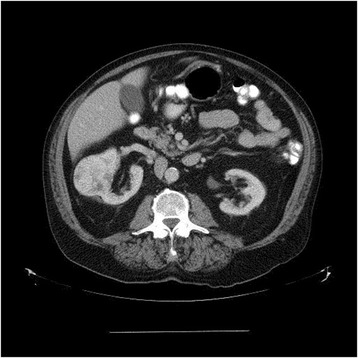
Contrast enhanced computed tomography of abdomen revealing solid enhancing mass arising laterally from right kidney

A 72 year old male with no previous history of malignancy was seen in the urology clinic at the local site for complaints of flank pain. On intravenous contrast enhanced computed tomography (CT) of the abdomen and pelvis, imaging revealed a 5.7 cm solid enhancing mass from the lateral aspect of the right kidney, suspicious for renal cell carcinoma (RCC) (Fig. 1). There was no renal venous invasion or pathologic retroperitoneal lymphadenopathy identified. Two months later, the patient underwent a right laparoscopic radical nephrectomy.

**Fig. 2:**
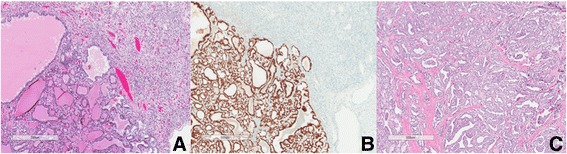
Clear cell renal cell carcinoma containing metastatic deposit of papillary thyroid carcinoma (left side of field; 2**a**). TTF1 immunostain highlights the focus of papillary thyroid carcinoma but is negative in the clear cell renal carcinoma (2**b**). Papillary thyroid carcinoma from right lobe of thyroid (2**c**)

Pathology report noted the right nephrectomy specimen contained a focally yellow well-circumscribed lesion on the upper pole. The histologic and immunohistochemical profile of this lesion, including positive for RCC marker, was typical for CCRCC. The tumor measured 5.5 cm in the greatest dimension and was limited to the kidney, giving a pathologic staging of pT1b and International Society of Urological Pathology (ISUP) Grade 2. Interestingly, the tumor contained a 3 mm focus of a lesion staining positive for Thyroid Transcription Factor-1 (TTF1), Thyroglobulin, and was negative for RCC marker (Fig. 2a, b). This finding led to the diagnosis of a tumor-to-tumor metastasis of PTC to CCRCC. The gross specimen was reexamined and further tissue was submitted, but no other foci were identified.

**Fig. 3:**
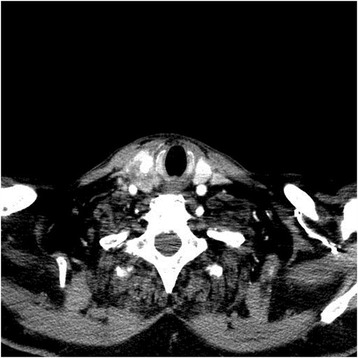
Contrast enhanced computed tomography of head and neck illustrating right thyroid mass

A subsequent ultrasound and CT of the head and neck revealed a heterogeneously hypodense 3.3 cm mass in the right thyroid lobe, with multifocal coarse and stippled calcifications, continuous with the overlying strap muscles (Fig. 3). There were also three right paratracheal nodes directly inferior to the mass, the largest measuring 6 mm. Given the confirmation of right sided thyroid mass on imaging and the immunohistochemistry proven focus of thyroid metastasis within the nephrectomy specimen, a preoperative FNA biopsy was not deemed necessary. The patient underwent a total thyroidectomy and level VI neck dissection. As indicated by imaging, there was extracapsular extension into the strap muscles and involvement of the first two tracheal rings posteriorly. Additionally, the lesion extended into the superior thyroid vein and lateral border of the internal jugular vein, which was excised after suture ligation. Intraoperative nerve monitoring demonstrated the recurrent laryngeal nerves were intact bilaterally.

Pathology revealed a classic variant multifocal PTC with two foci measuring 7.0 and 0.3 cm (Fig. 2c), a more accurate representation than the pre-operative CT Head and Neck which noted difficult visualization due to beam hardening artifacts from the shoulders. The level VI neck dissection yielded two ipsilateral lymph nodes positive for metastatic PTC, with no regional metastasis on the contralateral side. Ultimately, the thyroid specimen was positive for lymphatic vascular invasion, extrathyroidal extension with invasion of the tracheal cartilage. Therefore the tumor was staged T4aN1aM1. Postoperatively, the patient received radioiodine-131 ablation at a 150 mCi dose for remnant thyroid tissue ablation. The patient tolerated the therapeutic ablation without complications, and subsequent CT imaging and bone scan revealed no evidence of recurrent disease or metastasis. Thyroglobulin and TSH levels were 4081.0 pmol/L and 232.2 mIU/L respectively. On recent three month follow up the patient was recovering well and without signs of dysphagia or dysphonia. Laryngoscopy demonstrated mobile bilateral vocal cords.

## Discussion

PTC is most often diagnosed in middle aged females at a mean age of 31–49 years, and a female to male ratio between 2:1 and 3:1 [2]. Given the paucity of lifestyle, hereditary, and occupational risk factors for malignancy in our patient, the serendipitous detection of a late stage thyroid cancer metastasis most likely originated from a primary lesion that had grown for several years. Thyroid cancer accounts for only 1–2.5% of primary tumors metastasizing to the kidney, making a tumor-to-tumor metastasis from a thyroid cancer exceedingly uncommon [2]. Distant metastatic spread from the thyroid gland may be limited by the effect of the pulmonary filter, high partial pressure of oxygen in the gland, and iodine concentration in the thyroid [4]. Alternatively, the low rates of reported distant metastases may be due to an asymptomatic missed diagnosis, as autopsy reports of renal metastasis are 7.2% [1].

Tumor-to-tumor metastasis between the thyroid and kidney is an extremely rare occurrence, and report of RCC metastases from a WDTC has not yet been reported in the literature. The criteria for tumor-to-tumor metastasis between two independent primary neoplasms excludes collision tumors, spread of adjacent tumors, metastasis to hematopoietic tumors or mere tumor embolizations [3]. The underlying pathology has been hypothesized to be a result of either rich vascularity and perfusion to enable successful delivery and deposition of metastatic tumor cells, or a recipient cell microenvironment abundant in lipids and glycogen to create a favorable milieu for metastatic growth [3]. The former theory may explain the most common sites of WDTC metastasis to lung and bones at 50% and 26% respectively, from their robust vascular supply and proximity [2]. Both postulations may be concurrently in metastasis to lipid CCRCC, as the donor thyroid neoplasm is also well vascularized. Due to the generally indolent nature of the cancer, distant metastasis from a well differentiated thyroid tumor likely represents a late manifestation of the malignancy.

In both tumor-to-tumor metastasis and primary thyroid neoplasm metastasis to the kidney, the detection of renal metastasis may be challenging. Confounding factors include the inability of the metastatic lesion to trap iodine on iodine-131 scan, and difficulty differentiating between renal metastases and solid kidney tumors on MRI [1]. Similarly, in circumstances where a primary renal neoplasm is detected and a suspicious thyroid lesion is discovered in diagnostic imaging, differentiation of a primary versus secondary tumor may be difficult. A hypoechoic, non-homogeneous, and solitary appearance as well as cold radioiodine uptake tests are seen in both circumstances [5]. Hence fine needle aspiration (FNA) cytology should be employed to establish a preoperative diagnosis with high sensitivity and specificity, and ancillary studies such as immunohistochemistry can delineate primary versus metastatic disease.

While a dual population of cells on cytology may allude to an intra-tumor metastasis, immunohistochemistry can fortify a working diagnosis. Specific markers such as TTF1 which is found in the lung and thyroid gland, and thyroglobulin all support the classification of WDTC [1, 6].

Although both WDTC and CCRCC have a clear cell appearance, additional features such as multifocal growth, history of previous malignancies, and pattern of sinusoidal vascularization would suggest a secondary thyroid tumor [5]. The differential diagnosis should include primary tumors with clear cell features such as paraganglioma, papillary, follicular, and medullary carcinomas, metastasis most commonly from the lung, breast, and gastrointestinal tract, and tumor-to-tumor metastasis most frequently arising from a RCC [7]. In patients who have a suspected RCC and elevated thyroglobulin levels, both papillary and follicular thyroid carcinomas should be included in the differential diagnosis irrespective of solitary or unilateral renal masses, or negative history of thyroid cancer, as both secondary malignancy and tumor-to-tumor metastasis are possible [1, 6–8].

It is imperative to manage patients with distant metastatic disease from a primary WDTC with systemic iodine-131 ablation therapy, as up to 75% of these patient cohorts die within five years of diagnosis [8]. Factors such as age, primary tumor size, male gender, extrathyroidal extension, and both regional lymph node and distant renal metastasis may portend an unfavorable prognosis [2]. Given the average interval between primary thyroid carcinoma diagnosis and renal metastasis is 16.6 years, there is a broad period for possible intervention of this generally indolent disease [9].

## Conclusions

In summary, corroboration of diagnostic imaging findings with immunohistochemistry staining can consolidate a diagnosis of thyroid neoplasm tumor-to-tumor metastasis to a RCC, thereby prompting surgical excision. This is the first documented case of a WDTC metastasis to a known CCRCC.

## Abbreviations

CCRCC: Clear cell renal cell carcinoma
CT: Computed tomography
FNA: Fine needle aspiration
ISUP: International society of urological pathology
PTC: Papillary thyroid cancer
RCC: Renal cell carcinoma
TTF-1: Thyroid transcription factor-1
WDTC: Well-differentiated thyroid carcinoma
